# *IncucyteDRC*: An R package for the dose response analysis of live cell imaging data

**DOI:** 10.12688/f1000research.8694.1

**Published:** 2016-05-23

**Authors:** Philip J. Chapman, Dominic I. James, Amanda J. Watson, Gemma V. Hopkins, Ian D. Waddell, Donald J. Ogilvie

**Affiliations:** 1 Drug Discovery Unit, Cancer Research UK Manchester Institute, Manchester, UK

**Keywords:** R package, Drug discovery, Dose response curve, Live cell imaging, Oncology, Shiny

## Abstract

We present *IncucyteDRC*, an R package for the analysis of data from live cell imaging cell proliferation experiments carried out on the Essen Biosciences IncuCyte ZOOM instrument. The package provides a simple workflow for summarising data into a form that can be used to calculate dose response curves and EC50 values for small molecule inhibitors. Data from different cell lines, or cell lines grown under different conditions, can be normalised as to their doubling time. A simple graphical web interface, implemented using shiny, is provided for the benefit of non-R users. The software is potentially useful to any research group studying the impact of small molecule inhibitors on cell proliferation using the IncuCyte ZOOM.

## Introduction

Live cell imaging permits cell proliferation to be monitored in real time over a period of days or weeks, and the IncuCyte ZOOM from Essen Biosciences ( http://www.essenbioscience.com/essen-products/incucyte/) is a platform used for such experiments. In oncology drug discovery the proliferation of cancer cell lines can be monitored in the presence of different small molecule inhibitors at different concentrations to gain insight as to the efficacy and mechanism of action of novel compounds. This approach provides a useful alternative to measuring cell proliferation as a phenotypic endpoint after a fixed period such as 3 days. It has the advantage of being able to visualise graphically and morphologically when treatments impact on proliferative capacity. Furthermore, vehicle dosed cells can be used to control for population doubling resulting in normalisation across many different cell lines. This may be particularly important when downstream effects of compound treatment require a round of replication before they are apparent.

The IncuCyte ZOOM comes with sophisticated software ( http://www.essenbioscience.com/essen-products/software/incucyte-base-software/) for the analysis of imaging data and the calculation of metrics such as percent confluence, however we sought a more streamlined workflow for the analysis of data from cell-based compound screening assays. The result was the development of the R ^1^ package IncucyteDRC which permits quick and easy fitting of dose response curves and calculation of EC50 values, as well as the export of data to standard analysis packages such as GraphPad PRISM http://www.graphpad.com/scientific-software/prism/ and Dotmatics Studies http://www.dotmatics.com/products/studies/. In addition, a graphical user interface was developed as part of the package using Shiny ^2^, as well as a novel algorithm for normalizing data by cell doubling time.

## Methods

### Workflow overview

A basic workflow for the IncucyteDRC package is outlined below:

1. Import data and plate map information
2. Create an IncucyteDRCSet object
3. Fit growth curves to the data
4. Calculate the growth curve values at a given ‘cut time’
5. Carry out a dose response analysis using the extracted values

Optionally, the cut time can be calculated automatically for a required number of doublings.

### Data import

The importPlatemapXML function imports .Platemap files generated by the IncuCyte ZOOM software to define the contents of a plate. The function can extract the following parameters: compound (description, concentration and units), growth condition (description), and cell type (description, passage and seeding density). These are converted into a data.frame object which is used as a basis for further analysis. Alternatively, the importPlatemap function can import a data.frame or a tab delimited text file but these must be in the correct format - see example(importPlatemap).

The importIncucyteData function imports data generated by the Incucyte Zoom software, as described in detail in the package vignette entitled ’Exporting From Incucyte Zoom Software’. The output is a IncucyteDRCPlateData S3 object.

### Creating an IncucyteDRCSet object

The IncucyteDRCSet object is at the centre of the package workflow. Subsequent functions add to and operate on the data contained within the object. It can be initiated using the makeIncucyteDRCSet function and at its simplest combines a plate map data frame generated by importPlatemapXML function with an IncucyteDRCPlateData object created by the importIncucyteData function. In addition, a cut time can be specified, and a single row metadata data.frame provided.

A key attribute of an IncucyteDRCSet is that all data share a common control growth curve. Thus it is appropriate to have an IncucyteDRCSet object containing multiple compounds on a common cell line background, but if the cell line background changes then one object is needed per condition.

The splitIncucyteDRCPlateData function is a convenience function that splits up an IncucyteDRCPlateData object into a list of IncucyteDRCSet objects if necessary, or a single IncucyteDRCSet object if not. It also automatically populates the metadata element. Therefore it is sensible to use this function, rather than the makeIncucyteDRCSet function, in a standard workflow.

### Fit growth curves

The first step in the analysis process is to fit growth curves to the data using the base R loess function. This is done using the related functions fitGrowthCurvesGrouped and fitGrowthCurvesIndividual. The former combines replicates to create a single growth curve for a given sample and compound concentration, whilst the latter fits a growth curve for every single well on a plate. The IncucyteDRCSet object is updated with four dplyr ^3^ data frames (tbl_df objects) containing the models themselves and the fitted data for plotting. Refer to the dplyr ^3^ package documentation for more information on how models and other objects can be stored in data frames.

**Figure 1.**
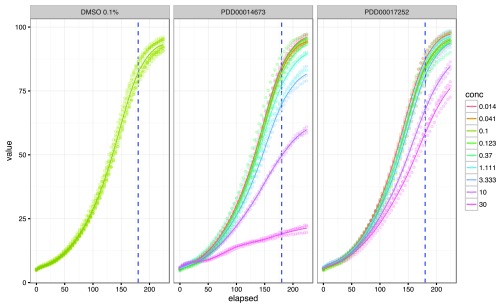
Visualisation of individual growth curves for the data in an IncucyteDRCSet object: x-axis is elapsed time in hours, y-axis is the growth value (% confluence in this case). The cut time is represented as a blue dashed vertical line.

Once the growth curves have been fitted, they can be visualised using the plotIncucyteDRCSet function ( Figure 1).

### Calculate growth curve values at a cut time

Although the growth curves themselves can give some insight into the effect that different compounds have on the growth of the cell line, to analyse this formally the growth parameter can be determined at a specific ‘cut time’ using the calculateDRCData function. This data is stored in the IncucyteDRCSet object and can subsequently be exported to various formats using the exportDRCDataToDataFrame, exportDRCDataToPRISM and exportDRCDataToDotmatics functions. These data can then form the basis of a dose response analysis to generate an EC50 value.

### Carry out a dose response analysis

It is advisable to export the dose response data and fit the curves in specialist software such as GraphPad PRISM or Dotmatics Studies, but for convenience there are also functions that utilise the drc package ^4^ to provide an end-to-end workflow. The fitDoseResponseCurve function generates models for each compound in the IncucyteDRCSet (using the drc::LL.4 function) and calculateEC50 predicts an EC50 value for each model (using the drc::EC50 function). The exportEC50Data function generates a data.frame containing the values stored in the IncucyteDRCSet object, whilst the dose response curves can be visualised with the plotDoseResponseCurve function.

**Figure 2.**
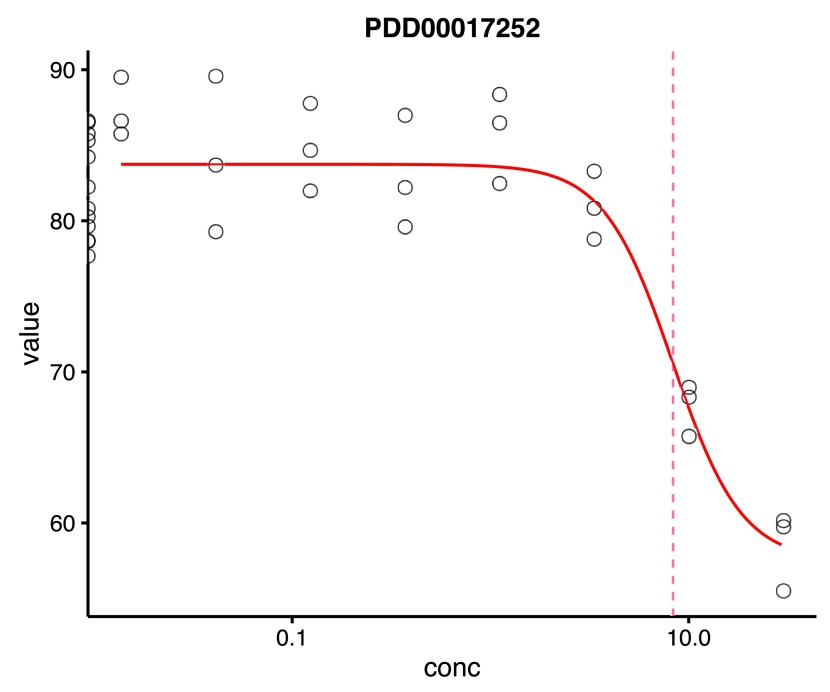
Visualisation of an individual dose response curve for a compound with the EC50 represented as a red dashed vertical line. The y-axis is the growth value (percent confluence in this case) whilst the x-axis is the micromolar compound concentration

### Calculation of the cut time

In the previous examples, a cut time has been manually provided following an inspection of the growth curves. Typically the cut time should be as long as possible to allow differences to become pronounced, but still during the exponential phase of cell growth before the cells become confluent. However, sometimes it might be desirable to determine the cut time for a given number of doublings, so that the EC50 for fast and slow growing cell lines can be normalised.

The calculateCutTimeForIDRCSet function can be run on an IncucyteDRCSet object to determine the optimal cut time from the control (vehicle treated) growth curves given a set of parameters. These include baseline_time (the timepoint to take as a baseline for calculating doublings), no_doublings (the number of doublings required) and max_val (the maximum allowable growth curve value). The algorithm works out the growth curve value at the baseline time and what value would correspond to a given number of doublings from this point, then returns the timepoint at which this value is achieved. If this value exceeds the maximum value, then the time at which the maximum value is returned instead, along with the actual number of doublings achieved.

In detail, the growth curve is truncated by removing its non-exponential phase along with any part exceeding the user defined maximum (usually 80%). The non-exponential phase is defined as the part of the curve after the timepoint at which the n=30 moving average of the second differences of the growth values reaches its minimum. This corresponds to the time at which the rate of cell growth is slowing most rapidly. The growth value required to reach the desired number of doublings from a user defined baseline time was calculated (using the predict function on the loess model object), and the elapsed time to reach this is returned as the cut time. If the required growth value is not reached on the truncated growth curve, then the maximum growth value achieved on the truncated curve is returned instead along with the actual number of doublings that occurred.

**Figure 3.**
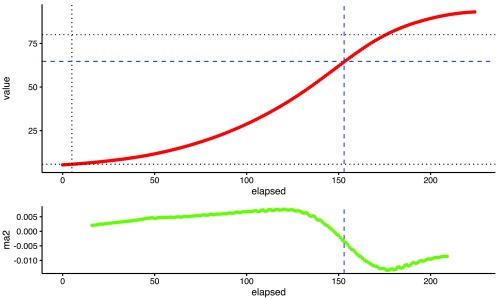
Control growth curve value (upper plot) and moving average of second differences (lower plot) used to calculate the cut time with elapsed time in hours on the x-axis. The black dashed lines represent user-defined parameters whilst the blue dashed lines are calculated by the algorithm. The blue vertical dashed line is the cut time.

### Operation

The IncucyteDRC R package is available on CRAN and is built on top of R version 3.2.3 ^1^ and Shiny 0.13.2 ^2^ and has no special R dependencies beyond packages that are available on CRAN and so should work on any operating system supported by R. A full list of R package dependencies is present in the Description file of the package.

The package can be installed from CRAN:

install.packages(’IncucyteDRC’)

The latest development version can be installed in an R session from GitHub using the devtools ^5^ package:

devtools::install_github(’chapmandu2/IncucyteDRC’)

Having successfully installed the IncucyteDRC package, it can be loaded using library(’IncucyteDRC’) and a detailed vignette is available which can be viewed by typing browseVignettes(’IncucyteDRC’). Help and examples are available for functions within the package e.g.

?importIncucyteData

example(importIncucyteData)

Finally, a Shiny app ^2^ provides a graphical user interface for the functionality within the package. This can be launched from within R using shinyVisApp() whilst details for how to set up the IncucyteDRC package as a Shiny app on a Shiny server is included in the vignette.

**Figure 4.**
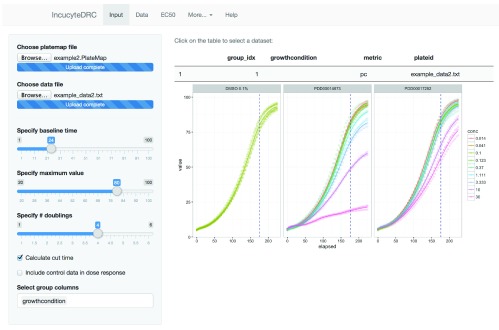
Screenshot of the Shiny web application that allows non-R users to run the IncucyteDRC workflow.

## Use cases

The package vignette contains detailed use cases using two example datasets. These can be loaded by running the examples for the importPlatemapXML and importIncucyteData functions:

example(’importPlatemapXML’)

example(’importIncucyteData’)

## Summary

The IncucyteDRC R package provides a simple, reproducible workflow for the dose response analysis of live cell imaging data from the IncuCyte Zoom instrument. To facilitate its use by non-R users, a comprehensive and user-friendly graphical interface is provided as a Shiny ^2^ web application. The software is freely available on CRAN and is potentially useful to any research group studying the impact of small molecule inhibitors on cell proliferation.

## Software availability

1. Software available from: https://cran.r-project.org/web/packages/IncucyteDRC/
2. Latest source code: https://github.com/chapmandu2/IncucyteDRC/
3. Archived source code as at time of publication: http://dx.doi.org/10.5281/zenodo.51260 ^6^
4. License: GPL-2
