# Supplementary material for: IncucyteDRC: An R package for the dose response analysis of live cell imaging data
Source: F1000Res. 2016 May 23;5:962. [Version 1] doi: 10.12688/f1000research.8694.1 (PMC5031130; doi:10.12688/f1000research.8694.1)
Supplement: Peer review history

# Peer review: Referee response for version 1

**DOI:** 10.5256/f1000research.9356.r16099

**Published:** 2016-09-16

**Authors:** Clara Alsinet-Armengol

**Affiliations:** 1 Wellcome Trust Sanger Institute, Hinxton, UK

The authors have developed an R package to visualise drug response data and make some basic calculations from experiments performed on the Incucyte ZOOM system. The funtions are easy to use and, interestingly, the useful IncucyteDRCSet object has been designed so that it can progressively integrate all data generated. Both the article and the R documentation are clearly explained.

 I would just make one suggestion on the manuscript. One of the features of the software is that it allows for normalization of the drug response by cell doubling time. A brief description of its relevance could be added.

I have read this submission. I believe that I have an appropriate level of expertise to confirm that it is of an acceptable scientific standard.

# Peer review: Referee response for version 1

**DOI:** 10.5256/f1000research.9356.r15204

**Published:** 2016-08-03

**Authors:** Guillaume Devailly

**Affiliations:** 1 Department of Developmental Biology, The Roslin Institute, University of Edinburgh, Edinburgh, UK

P. Chapman *et al.* developed an R package to analyse data obtained from the Incucyte instrument, and specifically growth curve responses to various treatment. While one could interpret Incucyte data without this package, or without any programming skills at all, there is no doubt that Incucyte users knowing R, or intensive Incucyte users wishing to automatize their analysis, will greatly benefit from using it. Overall the package is working well, is easily available through the popular CRAN repository, is well documented, with notably nice vignette, a video tutorial and a companion shiny app. The present manuscript present this package appropriately.

 I have no major concern with it, and only small comments and suggestions for improvement.

 About the manuscript:

1. Package availability on CRAN could be mentioned in the abstract.
2. Authors could detail the reasons why they advise against using their package for carrying dose response analysis, in favour of alternative software.
3. Page 2: “A  key  attribute  of  an  IncucyteDRCSet  is  that  all  data  share  a common  control  growth  curve.” I was a bit confused at first as I interpreted this as the non-support of replicates for the negative control. It could be mentioned that the control growth curve can (and should) come from multiple replicated control wells.
4. Since the public shiny app is now available, it could be mentioned in the manuscript, and even in the abstract, if the authors are willing to support it for a few years.

About the package (I tested only the CRAN version IncucyteDRC_0.5.4, what follows might not apply to future version, or to even to the github version.):

1. The importPlatemap and importPlatemapXML functions has a default value “DMSO” as a control_cpd parameter. I feel this is a terrible idea as users might at first not be aware of this crucial parameter. Not everyone use DMSO as a control, and some silly users might even have named it “dmso” in their incucyte data. Notably it is not used (left as default) in the first usage of the importPlatemapXML function in the “Overview of the IncucyteDRC Package” vignette. I would advise to not use any default value for this parameter and throw an error if it is missing.
2. The importIncucyteData helps mentioned that you wanted to add little more details about it, you might want to have a look (“NEED MORE DOCUMENTATION HERE ABOUT HOW TO DO EXPORT!!”).
3. The help pages for functions might benefit form a “suggest/see also” section (i.e. linking back the various import and export functions).
4. The IncucyteDRCSetList object is quite useless at the moment. Could you overload the fitGrowthCurvesGrouped, fitGrowthCurvesIndividual, calculateCutTimeForIDRCSet, calculateDRCData, fitDoseResponseCurve, and calculateEC50 function so that when provided with an IncucyteDRCSetList object, they return an IncucyteDRCSetList object in which each IncucyteDRCSet object has been modified? That would be a bit more convenient for the user than the apply method you suggest in the vignette.
5. I got an error with the local version of the shiny app when testing it with the example 2 files (Error in match.arg: 'arg' must be NULL or a character vector). Since it does not affect the shinyapps.io version, I guess it might be already fixed in github, or it might be a local issue.

I have read this submission. I believe that I have an appropriate level of expertise to confirm that it is of an acceptable scientific standard.
